# EchiNam: multicenter retrospective study on the experience, challenges, and pitfalls in the diagnosis and treatment of alveolar echinococcosis in Belgium

**DOI:** 10.1007/s10096-024-04996-4

**Published:** 2024-11-25

**Authors:** Pierre-Emmanuel Plum, Nathalie Ausselet, François Kidd, Séverine Noirhomme, Maria-Grazia Garrino, Alexandra Dili, Marie-Pierre Hayette, Olivier Detry, Philippe Leonard, Christian Motet, Maya Hites, Marc Bourgeois, Isabel Montesinos, Bénédicte Delaere

**Affiliations:** 1 Department of Infectious Diseases, CHU UCL Namur (Site Godinne), Université catholique de Louvain, Avenue Dr Gaston Thérasse 1, Yvoir, 5530 Belgium; 2 Department of Infectious Diseases, CHU UCL Namur (Site Namur), Université catholique de Louvain, Namur, Belgium; 3 Department of Infectious Diseases, Centre Hospitalier Régional de Namur (Site Namur), Namur, Belgium; 4 Department of Clinical Microbiology, Centre Hospitalier Régional de Namur (Site Namur), Namur, Belgium; 5 HPB Surgery Unit, CHU UCL Namur (Site Godinne), Université catholique de Louvain, Yvoir, Belgium; 6 National Reference Laboratory for Echinococcosis, Department of Clinical Microbiology, University Hospital of Liège (CHU Liège), Liège, Belgium; 7 Department of Abdominal Surgery and Transplantation, University Hospital of Liège (CHU Liège), Liège, Belgium; 8 Department of Infectious Diseases, University Hospital of Liège (CHU Liège), Liège, Belgium; 9 Clinic of Infectious Diseases, Hôpital Universitaire de Bruxelles (H.U.B), Université Libre de Bruxelles, Brussels, Belgium; 10 Department of Clinical Microbiology, CHU UCL Namur (Site Godinne), Université catholique de Louvain, Yvoir, Belgium

**Keywords:** Alveolar echinococcosis, Challenges, Pitfalls, Diagnosis, Treatment, Belgium

## Abstract

**Objectives:**

The aim of this retrospective study was to collect epidemiological, clinical, laboratory, imaging, management, and follow-up data on cases of alveolar echinococcosis (AE) diagnosed and/or followed up within the Namur Hospital Network (NHN) in order to gather information on the challenges, pitfalls, and overall experience in the diagnosis and treatment of AE.

**Methods:**

EchiNam was a multicenter retrospective study. Patients diagnosed and/or treated for probable or confirmed AE in the NHN between 2002 and 2023 were included in the study. Patient selection was based on diagnosis codes, laboratory results, and albendazole (ABZ) dispensing.

**Results:**

A total of 22 AE cases were retrieved, of which four were classified as probable and 18 as confirmed cases. Nine patients were either asymptomatic or had symptoms attributed to another disease. Clinical examination yielded pathologic findings in 10 patients. The median duration from the first AE-suggestive laboratory abnormalities to diagnosis was 176 days, and the median duration from the first AE-related imaging abnormalities to diagnosis was 133 days. Overall, 12 patients underwent surgical resection, with only four achieving complete lesion resection. Nine patients experienced ABZ-related adverse effects, with temporary ABZ discontinuation in five.

**Conclusion:**

Due to various factors such as a long incubation period and a lack of awareness among Belgian physicians, AE is often diagnosed at advanced disease stages. Treatment then becomes more complex or even suboptimal, resulting in prolonged therapy, higher risk of adverse effects, significantly impaired quality of life, poor prognosis, and higher mortality rates. Measures should be taken to achieve early diagnosis in endemic areas.

**Supplementary Information:**

The online version contains supplementary material available at 10.1007/s10096-024-04996-4.

## Introduction

Alveolar echinococcosis (AE) is a rare and neglected zoonosis caused by the larval stage of the fox tapeworm *Echinococcus multilocularis* (EM) [1–3].

The parasite is endemic in North America (north-west Alaska, north-west Canada, and central northern states of the United States of America), northern, eastern, and central Europe (which has been a known core highly endemic area for AE since the end of the 19th century), and Asia (with a highly endemic region covering most of Russia, central Asia [Kazakhstan and Kyrgyzstan], western China [with a high incidence of AE on the Tibetan plateau], eastern Turkey, and the northern Japanese island of Hokkaido) [1, 2, 4, 5]. The global incidence is estimated at 18,235 new cases of AE per year, 91% of which occur in China [4].

AE is considered rare in European countries where the disease is present, with an estimated average incidence of 0.03–0.20 cases/100,000 inhabitants/year [6]. Prevalences in highly endemic areas of central Europe are estimated to be between 2 and 40 per 100,000 inhabitants [7]. Studies have shown an expansion of the central European endemic area of AE to the north, west, and east [5]. Human cases have been detected in countries (Czech Republic, Hungary, Netherlands, Slovakia, Slovenia, Romania) considered free of AE before 1995, and the incidence has doubled in the endemic areas of France, Switzerland, Germany, and Austria [1, 5, 6]. The expansion of AE in Europe is due to several factors: increasing red fox populations in both rural and urban areas (due to rabies eradication, elimination of fox predators, reduced hunting pressure, abundant availability of anthropogenic food, and fox feeding) [7, 8]; more frequent human–fox interactions (due to positive human attitudes toward foxes that promote colonization of residential areas) [8]; increasing EM infections in European fox and dog populations [8, 9]. Sixteen European countries reported at least one confirmed case of AE to the European Centre for Disease Prevention and Control between 2018 and 2022: Austria, Belgium, Croatia, Czech Republic, Estonia, France, Germany, Hungary, Italy, Latvia, Lithuania, Norway, Poland, Slovakia, Slovenia, and Sweden. The majority of reported European AE cases are clustered in the Swiss Jura, northeastern Switzerland, southern Germany, western Austria, central France, French Jura, and Savoy [10]. In terms of new cases, many European regions may not yet have reached a plateau [11].

The first Belgian autochthonous case of AE was described in 2002 by Delbecque et al. in a patient living in the southern part of the province of Liège [12]. In Belgium, AE is considered a rare disease and its reporting is not mandatory. In 2022, 12 cases were reported by the Belgian National Reference Laboratory (BNRL) [13]. Endemic cases of AE are unevenly distributed across the country: They are more common in Wallonia, the southern part of Belgium, including the three districts of Namur, Liège, and Luxembourg [3, 13, 14], where the red fox population is increasing and several of them are infected with EM [15, 16]. The overall proportion of EM infected foxes is higher in Wallonia (38.6%) than in Flanders (2.1%) [15, 17]. The highest proportions of infected foxes in Wallonia are in Ardenne (42.2%) and Hesbaye (42.1%), then in Fagne-Famenne (38.5%), Plateau de Herve (30.8%), Condroz (29.2%), and finally Lorraine (26.9%). These data have not yet been published, but were presented in 2023 by A. Linden et al.. The impact of rodent density on the infection rate in foxes has already been demonstrated by Saitoh et al. [18]. A study carried out in the French Ardennes showed that the increasing rodent population density could explain the rising proportion of infected foxes in this part of northern France [19]. A similar phenomenon is probably taking place in the neighboring Belgian Ardennes, but this remains to be demonstrated.

*E. multilocularis* is a small tapeworm that reproduces via a sylvatic cycle based on predation relationships between the definitive host, foxes, and intermediate hosts consisting mainly of cricetids (particularly *Microtus* sp. and *Arvicola terrestris*) [1–3, 7, 19]. An urban cycle has also been described, with dogs and, less frequently, cats acting as definitive hosts [1]. Humans are accidental intermediate hosts of this parasite. They are typically infected via the fecal-oral route, usually by ingestion of EM eggs [1].

Identified risk factors for AE infection are mainly owning dogs and cats, having a kitchen garden, being a farmer, visiting forests for professional purposes, and hunting [20].

AE has a very long incubation period (5 to 15 years), with the metacestode slowly increasing in size, mimicking a malignancy as it invades and destroys neighboring tissues and organs. In advanced stages of the disease, extrahepatic locations are also possible [1, 6, 21]. The malignant behavior of the metacestode is responsible for the high mortality associated with AE, especially in advanced stages, when patients are inadequately diagnosed and treated [11, 22]. AE is fatal in 90% of untreated patients within 10 years of diagnosis [23]. A French study showed that, when treated, the mortality of AE cases tended to merge with that of the general population matched for sex, age, and calendar year [24].

AE cases are classified by the World Health Organization Informal Working Group on Echinococcosis (WHO-IWGE) as possible, probable, or confirmed. Diagnosis of AE is based on the combined results of epidemiological and risk factor investigation, serology, imaging examinations, pathology, and molecular methods (i.e., polymerase chain reaction [PCR]) [1]. The diagnostic performance of *Echinococcus* PCR varies from 69.8 to 100% depending on the PCR method and sample quality [25]. The specificity depends on the DNA target and can reach 100%, allowing species differentiation between *E. multilocularis* and *Echinococcus granulosus* [26, 27]. Imaging modalities, mainly fluorodeoxyglucose (FDG) positron emission tomography (PET) with delayed image acquisition and magnetic resonance imaging (MRI), allow the stratification of AE lesions as active or inactive and the classification of the disease according to the WHO-IWGE guidelines [1, 28–30]. This classification provides essential information to clinicians, enabling them to set up a stage-based multidisciplinary approach to manage AE cases [30, 31]. Nowadays, FDG-PET is considered a key high-performance examination for AE diagnosis, as mentioned above, and for follow-up [1, 32].

Current guidelines recommend complete resection of the parasitic lesion(s) with a safety margin, if possible, followed by a 2-year postoperative antiparasitic therapy with oral albendazole (ABZ) at a dosage of 15 mg/kg/day [1, 28]. Because liver allotransplantation can be technically difficult with a high risk of postoperative local and distant recurrence, this surgery is considered a last resort treatment for patients with very symptomatic AE and inoperable lesion [33]. If radical resection has not been performed, prolonged ABZ suppressive treatment is recommended, as studies have suggested that this treatment may inhibit the growth of parasitic masses and induce regression of AE lesions [1, 2, 34, 35]. The algorithm of Hao Wen et al. suggests an annual Em18 serological follow-up, with FDG-PET being performed every 2 years. A decision of discontinuing ABZ treatment is made after two consecutive negative tests [1]. ABZ treatment may cause major or minor adverse effects [14, 34]. According to the Food and Drug Administration (FDA), very common (≥ 10% of patients) adverse effects include elevated liver enzymes and headache. Common (1–10%) adverse effects include increased intracranial pressure, dizziness, abdominal pain, nausea, vomiting, reversible alopecia, fever, and leukopenia. There is currently no equally effective pharmaceutical alternative to ABZ [1]. Due to the pharmacokinetics of ABZ, monitoring of ABZ sulfoxide plasma levels is necessary to adjust the drug dose [28]. ABZ treatment in this indication has been reimbursed in Belgium since September 2017 [36].

In the European endemic regions, prolonged ABZ treatment in patients who could not undergo radical surgical resection has led to the transformation of AE from a fatal disease to a chronic pathology. As a result, the quality of life of affected patients can be significantly impaired due to various treatment- and disease-related complications such as ABZ adverse effects, biliary obstruction, septicemia, portal hypertension, and Budd–Chiari disease [11]. Current estimates suggest an average of 666,434 disability-adjusted life years (DALYs) associated with this disease worldwide [4].

As AE is rare in Belgium, studies describing series of autochthonous Belgian cases remain scarce. This study sought to collect epidemiological, clinical, laboratory, imaging, management, and follow-up data on AE cases diagnosed and/or followed up within the Namur Hospital Network (NHN) in order to gather information on the challenges, pitfalls, and overall experience in AE diagnosis and treatment.

## Materials and methods

EchiNam was a multicenter retrospective study that included probable or confirmed AE cases diagnosed and/or followed within the NHN between 2002 and 2023. A map of Belgium showing the NHN sites is available as supplementary material (Fig. 1).This study was approved by the ethics committee of the CHU UCL Namur University Hospital (reference number: B0392022000108).

Informed consent of patients was not required, as the study was an anonymized retrospective study based exclusively on data already available in the electronic medical records.

Patients were selected from the databases of the five NHN hospitals [1667 acute care beds (CHU UCL Namur (sites Godinne, Namur, Dinant), Clinique Saint-Luc Bouge, and Centre Hospitalier Régional de Namur (site Namur)] as follows:

1. Retrieval of patients with a putative AE diagnosis between 2002 and 2023, based on available data: ICD-9 and ICD-10 diagnosis codes, hospital pharmacy data (ABZ supply between 2002 and 2023), and microbiology results (positive *Echinococcus* serology or positive EM PCR) from local laboratories and the BNRL. Prior to June 2021, AE serology and PCR testing were performed by different laboratories using different methods. Since then, the CHU Liège has been the BNRL for echinococcosis in Belgium, where all *Echinococcus* confirmation serologies and PCR tests are performed for the NHN.
2. Based on WHO-IWGE criteria, patients classified as probable AE cases (serological, imaging, epidemiological, and clinical findings suggestive of AE) or confirmed AE cases (pathological or molecular biological confirmation of AE) [1] were included in the study.
3. Epidemiological, clinical, laboratory, imaging, surgical, treatment, and follow-up data were collected from the electronic medical records in 2023.
  Blood test parameters considered suggestive of AE were leukocytosis, elevated C-reactive protein, elevated eosinophil count, or elevated liver enzymes with no other identified etiology [37].
  Based on imaging (mainly FDG-PET and MRI), patients’ disease was classified according to WHO-IWGE guidelines [1, 38].
  The quality of surgical lesion resection was classified as follows:
   ● R0: Complete lesion resection with no residual disease;
   ● R1: Microscopic residue with less than 1 mm margin;
   ● R2: Macroscopic residue.
  The therapeutic range for monitoring of ABZ sulfoxide plasma levels was 0.28–0.84 mg/l [28].

4. Data were pseudonymized in a password-protected Excel file.
5. Statistics were mainly descriptive, using medians, means, and confidence intervals to summarize data, as the number of patients included was too small to perform univariate and multivariate analysis.

## Results

Based on WHO-IWGE criteria, a total of 22 AE cases were selected, including four probable and 18 confirmed cases. Regarding their medical conditions, 32% (*n* = 7) of patients were immunosuppressed, including those with progressive solid or hematologic cancers and those treated with immunosuppressive drugs. The epidemiological and clinical characteristics of patients are described in Table 1.

**Table 1:** Demographic characteristics and medical conditions of the 22 patients

General information, *n* (%)	
Median age at diagnosis (years) (95% CI)	67 (55.1–68.7)
Male; sex ratio	14 (63); 175
At least one underlying medical condition, *n* (%)	15 (68)
Smoking	6 (27)
Chronic alcoholism	4 (18)
Diabetes	3 (13.5)
Chronic renal failure	3 (13.5)
Cirrhosis	3 (13.5)
Ischemic cardiopathy	2 (9)
Hypertension	2 (9)
Chronic obstructive pulmonary disease	2 (9)
Sarcoidosis	2 (9)
Stroke	1 (4.5)
Immunosuppression	7 (32)
Immunosuppressive treatment	3 (14)
Hematologic cancer	2 (9)
Solid cancer	2 (9)

Exposure risk factors are described in Table 2. Three patients did not have their specific risk factors documented, except for occupational exposure.

**Table 2:** Exposure risk factors for AE available in medical records of 19 patients

Risk factors, *n* (%)	
Dog ownership	12 (63)
Picking or eating mushrooms or berries	11 (58)
Having a kitchen garden	9 (47)
Forest visits for non-occupational purposes	6 (31.5)
Cat ownership	6 (31.5)
Forest visits for professional purposes	4 (21)
Farmer	3 (16)
Risk factor count, *n* (%)	
No risk factor	1 (5.5)
One risk factor	2 (10.5)
At least two risk factors	16 (84)

In our cohort, no AE cases were reported within the household.

Thirteen patients had symptoms attributed to AE at the time of diagnostic work-up. Nine patients were asymptomatic or had symptoms attributed to another disease.

Symptoms and clinical findings at the time of diagnostic work-up are described in Table 3.

The median duration of AE-related symptoms prior to diagnostic work-up was 60 days (0–3650 days) for 11 patients; duration was not available for two pauci-symptomatic patients. The median duration from first laboratory abnormalities to diagnosis was 176 days (2–783 days) for the 14 patients with abnormal tests attributed to AE. Laboratory test results are summarized in Table 4. 

**Table 3:** Symptoms and clinical findings at the time of diagnostic work-up, AE imaging and staging at the time of diagnostic work-up, AE-associated complications of the 22 patients

Symptoms	
Asymptomatic or symptoms attributed to another disease, *n* (%)	9 (41)
Symptoms attributed to AE, *n* (%)	13 (59)
Systemic symptoms, *n* (%)	11 (84.5)
Asthenia	8 (73)
Weight loss	7 (63.5)
Symptoms related to hepatic or perihepatic invasion, *n* (%)	10 (77)
Abdominal pain	9 (90)
Jaundice	3 (30)
Cough	3 (30)
Vomiting	1 (10)
Dyspnea	1 (10)
Nausea	1 (10)
Dyspepsia	1 (10)
Clinical findings	
Normal clinical examination or pathological clinical examination due toanother disease, *n* (%)	12 (54.5)
Pathological clinical examination attributed to AE, n (%)	10 (45.5)
Hepatomegaly	6 (60)
Jaundice	3 (30)
Pathological pulmonary auscultation	3 (30)
Pain on abdominal palpation	2 (20)
Ascites	1 (10)
Imaging and staging	
Liver involvement, *n* (%)	22 (100)
Right lobe only	12 (54.5)
Left lobe only	4 (18)
Two lobes	6 (27)
Median largest lesion size at diagnosis (mm)	65 (50–85)
Extension beyond the liver capsule, *n* (%)	8 (36.5)
Metastatic disease at diagnosis, *n* (%)	1 (4.5)
PET scans with significant FDG uptake in the periphery of parasitic lesions, *n* (%)	20 (91)
WHO-IWGE classification, *n* (%)	
I	5 (22.5)
II	4 (18)
IIIa	1 (4.5)
IIIb	5 (22.5)
IV	7 (32)
Complications	
At least one complication, *n* (%)	12 (54.5)
Bacterial superinfection of the parasitic lesion	1 (8,5)
Angiocholitis	1 (8,5)
Biliary compression	11 (91.5)
Percutaneous biliary drainage	1 (9)
Cysto-biliary fistula	2 (16.5)
Veinous compression	8 (66.5)
Portal or mesenteric thrombosis	3 (37.5)
Imaging sign of hypertension	1 (33)
Endoscopic variceal ligation for bleeding esophageal varices	1 (100)

**Table 4:** Laboratory tests performed during the diagnostic work-up of the 22 patients

Nonspecific laboratory tests	
Normal tests, *n* (%)	4 (18)
Abnormal tests, *n* (%)	18 (82)
Abnormal tests attributed to underlying disease(s) other than AE, *n* (%)	4 (22)
Abnormal tests attributed to AE, *n* (%)	14 (78)
Median C-reactive protein level (mg/l) (95% CI)	6.45 (2.9–15)
C-reactive protein level > 8 mg/l, *n* (%)	4 (28.5)
Liver enzymes	
Abnormal liver enzymes, *n* (%)	13 (93)
Cholestasis, *n* (%)	5 (38.5)
Cytolytic hepatitis, *n* (%)	1 (7.5)
Mixed, *n* (%)	7 (54)
Median blood eosinophil count (cells/mm³) (95% CI)	245 (121–555)
Blood eosinophil count > 500 cells/mm³, *n* (%)	2 (14)
Immunodiagnosis, *n* (%)	
Serologic screening performed	21 (95.5)
Positive serologic screening	15 (71.5)
Negative serologic screening	3 (14.5)
Serologic screening result in the grey zone	3 (14.5)
Western blots performed	15 (68)
Positive for *Echinococcus* sp.	15 (100)
Results suggestive of AE	11 (73.5)
Molecular diagnosis n (%)	
PCR performed	10 (45.5)
Positive PCR results	8 (80)

The median duration from first pathologic imaging suggestive of echinococcosis to confirmation of diagnosis was 133 days (2–4811 days).

In this cohort, AE diagnostic work-up was initiated because of nonspecific laboratory findings (leukocytosis, elevated C-reactive protein, elevated eosinophil count, or elevated liver enzymes) or imaging incidentalomas in 11 patients, while diagnostic work-up was initiated because of AE-related symptoms in the remaining 11 patients.

In 16 patients, echinococcosis was not part of the differential diagnosis suggested by the radiologists at the time of initial imaging.

With respect to imaging and staging, the results are summarized in Table 3.

According to the WHO-IWGE classification, three immunocompromised patients were classified as stage I, two as stage II, one as stage IIIb, and one as stage IV. Non-immunocompromised patients (*n* = 15) were classified as follows: two patients stage I, two stage II, one stage IIIa, four stage IIIb, and six stage IV.

Serologic screening (i.e., ELISA, indirect hemagglutination, or immunodiffusion) was performed in all but one patient. The results are shown in Table 4. In immunocompromised patients (*n* = 7), serologic screening results were as follows: two patients had negative results, one had inconclusive results, and four had positive results.

Western blots (WB) were performed in 15 patients (68%). The results are presented in Table 4. All WBs were positive for *Echinococcus* sp., including in three patients with negative or inconclusive serologic screening results.

In immunocompromised patients (*n* = 7), WB results were as follows: three patients had no WB performed, zero had negative results, and four had positive results (two were positive for *Echinococcus* sp. and two were suggestive of AE).

Four patients (18%) underwent echo-endoscopic biopsy for AE diagnosis, but only one patient had confirmatory histology (25%). PCR was negative in the one patient who underwent this test. Eleven patients (50%) underwent percutaneous biopsy: histology was consistent with AE in seven patients (63.5%), while PCR was positive in seven of eight patients (87.5%) who underwent this test. In two cases, a laparoscopy was performed for diagnosis and evaluation purposes. In these patients, the histopathology results were consistent with AE, while PCR was positive in the patient who underwent this test.

Twelve patients (54.5%) developed AE-associated complications (Table 4).

Curative intent surgery was performed in 12 patients (54.5%). Details are described in Table 5.

**Table 5:** Surgery for AE lesion resection

	*N* = 22 (%)	Year of surgery
Surgical lesion resection, *n* (%)	12 (54.5)	
Preoperative diagnosis, *n* (%)		
AE as the primary suspected diagnosis at the time of surgery	8 (66.5)	
Preoperative misdiagnosis of neoplasia	4 (33.5)	
Quality of AE lesion resection, *n* (%)		
R0	4 (33.5)	2014, 2014, 2019, 2020
R1	6 (50)	2013, 2013, 2016, 2017, 2017, 2021
R2	2 (16.5)	2013, 2019
Postoperative complications, *n* (%)	8 (66.5)	
Non-severe complications	3 (37.5)	
Severe complications	5 (62.5)	
Liver failure	2 (40)	
Death (hemorrhagic shock)	1 (20)	
Massive pulmonary embolism	1 (20)	
Acute renal failure requiring dialysis	1 (20)	
Acidosis	1 (20)	
Biliary fistula with infected bilioma	1 (20)	
No surgical resection, *n* (%)	10 (45.5)	
Non-resectable lesions	6 (60)	
Contraindication to surgery	3 (30)	
Patient refused surgery	1 (10)	
Non-evolutive disease	1 (10)	
Patient scheduled for surgery at time of data collection	1 (10)	

All the patients operated on (*n* = 12) had an AE-compatible result from pathology examination, while eight patients (66.5%) underwent EM PCR testing that turned out to be positive.

Except the patient who died during surgery, all patients (*n* = 21) received ABZ treatment. At the time of data collection, 10 patients were still receiving ABZ treatment and 10 were not. Data were missing for one patient (loss to follow-up). Adverse effects attributed to ABZ are summarized in Table 6.

**Table 6:** Albendazole treatment in 21 patients

Albendazole (ABZ) treatment, *n* (%)	21 (95)
At least one adverse effect attributed to ABZ treatment, *n* (%)	9 (43)
Non-severe	8 (89)
Increased liver enzymes	2 (25)
Nausea	2 (25)
Dyspepsia	2 (25)
Abdominal pain	1 (12.5)
Polyneuropathy	1 (12.5)
Dysgeusia	1 (12.5)
Anemia	1 (12.5)
Alopecia	1 (12.5)
Severe	1 (12.5)
More than tenfold increase in liver enzymes	1 (12.5)
Temporary discontinuation of ABZ treatment, *n* (%)	8 (38)
Due to adverse effects attributed to ABZ treatment	5 (62.5)
Increased liver enzymes	2 (40)
Polyneuropathy	1 (20)
Abdominal pain	1 (20)
Anemia	1 (20)
Leukopenia	0 (0)
Due to ABZ availability (reimbursement issue or stock shortage)	3 (37.5)
ABZ treatment discontinuation, *n* (%)	10 (47.5)
Imaging stability for 4 or 5 years after initiation of postoperative ABZ treatment	2 (20)
Imaging stability for 4 years after initiation of ABZ treatment with initial non-significant FDG lesion uptake on PET scan	1 (10)
Completion of 2 years of postoperative ABZ treatment	4 (40)
Death	3 (30)
ABZ sulfoxide monitoring, *n* (%)	15 (71.5)
Within therapeutic range (0.28–0.84 mg/l) on first test	5 (33.5)
Overdosing on first test	6 (40)
Underdosing on first test	4 (26.5)
Overdosing and non-severe ABZ-attributed adverse effects	4 (26.5)
Overdosing and severe ABZ-attributed adverse effects	1 (6.5)

Table 7 describes ABZ treatment according to lesion resection quality.

**Table 7:** Albendazole treatment in operated patients correlated to lesion resection quality in 12 patients

Surgery, *n* (%)	12 (55)
R0 surgery, *n* (%)	4 (33.5)
Postoperative ABZ treatment discontinued after 2 years	3 (75)
Death within 2 years of surgery with ABZ treatment ongoing at time of death	1 (25)
R1 surgery, *n* (%)	6 (50)
Postoperative ABZ treatment stopped due to stability on follow-up imaging (PET scan and MRI)	3 (50)
After 2 years	1 (33.5)
After 4 years	1 (33.5)
After 5 years	1 (33.5)
Death during surgery	1 (16.5)
ABZ treatment ongoing at time of the study	2 (33.5)
R2 surgery, *n* (%)	2 (16.5)
ABZ treatment ongoing at time of the study	2 (100)

Initial disease progression was observed on imaging in three patients despite ABZ treatment, but only one had initial symptoms due to disease progression (pleural effusion responsible for dyspnea, treated with surgical paracentesis). One of these three patients was immunocompromised. According to the WHO-IWGE classification, the two non-immunocompromised patients were classified as stage II and IV at diagnosis. The immunocompromised patient was classified as stage I. Two of these three patients showed stability in lesion size after initial disease progression. The third patient died during follow-up. The remaining 19 patients remained stable or experienced a reduction in lesion size under treatment. One patient was lost to follow-up, 22 months after diagnosis.

Overall, five of 22 patients died during the study period, but only one death was considered AE-related (hemorrhagic shock during curative intent surgery).

The median duration from diagnosis to death was 395 days (95% CI: 294–874) for the five patients who died during the study period. The median duration from curative intent surgery to death was 465 days (95% CI: 52–1213) for the four patients who underwent surgery and died during the study period. The duration from ABZ treatment discontinuation (by physician order) to death was 743 days for the one patient who died after ABZ treatment discontinuation. The overall survival rate at 2 years after diagnosis (patients with at least 2 years of follow-up) was 14/18 (78%). The overall survival rate at 5 years after diagnosis (patients with at least 5 years of follow-up) was 10/14 (71%). The survival rate at 2 years after surgery for operated patients (follow-up of at least 2 years) was 9/12 (75%). The survival rate at 5 years after surgery for operated patients (follow-up of at least 5 years) was 6/10 (60%). The survival rate at 2 years after ABZ treatment initiation for non-operated patients (follow-up of at least 2 years) was 3/4 (75%). The survival rate at 5 years after ABZ treatment initiation for non-operated patients (follow-up of at least 5 years) was 2/3 (66%).

## Discussion

According to the BNRL, the detection of AE cases in Belgium has been increasing since 2010 and currently stands at around 10 to 12 cases per year. This increase can be attributed to several factors (as described in the introduction), but also to changes in the reporting method. From 2021, the new BNRL reporting system has become more comprehensive, requiring all Belgian laboratories to fill in a detailed form.

The BNRL practices are described as supplementary material.

As the first Belgian autochthonous case of AE was described only 20 years ago and a limited number of cases have been declared since then, the overall Belgian experience with AE is rather limited compared to highly endemic European countries (e.g., France, Germany, Switzerland) [12]. In addition, there are no national guidelines for AE management in Belgium. For these reasons, strategies for the diagnosis and management of AE in Belgium are currently based on international recommendations [1, 28].

New diagnoses and difficult AE cases are discussed with the Belgian National Reference Center (which includes hepatologists, infectious disease specialists, surgeons, microbiologists, and BNRL members with expertise in the management of AE cases) located at the University Hospital of Liège and, if necessary, with the French National Reference Center located at the University of Franche-Comté.

### Epidemiology

In our study, the median age at diagnosis was 67 years and approximately 32% of patients were immunosuppressed, which is consistent with observations in the CHU Liège cohort [14]. The most common exposure risk factors observed in our population were dog ownership and mushroom or berry picking.

In agreement with Yang et al. [39]., no cases were reported in the household of our patients. However, such cases were not actively sought in all patients, probably due to limited knowledge of AE risk factors among Belgian physicians and, as AE reporting is not mandatory in Belgium, to a lack of systematic inquiry by health authorities among persons living in the household and in close proximity to AE cases [40].

### Diagnosis

Delayed diagnosis was a key issue in this cohort and was mainly due to a lack of awareness of AE among Belgian physicians [40].

Symptoms and clinical findings may falsely reassure or mislead clinicians, as 41% of patients were asymptomatic at the time of diagnostic (or had symptoms attributed to another disease) and the majority of patients had normal clinical examination (or pathological clinical examination due to another disease). This may explain the delay between initial symptom onset and diagnostic work-up, such as laboratory tests and imaging. The median duration in this study was 60 days.

In our cohort, 36.5% of patients had either normal blood tests or laboratory abnormalities attributed to their comorbidities, falsely reassuring clinicians and delaying diagnostic work-up.

The median duration from first detection of imaging or non-specific laboratory abnormalities associated with AE to definitive diagnosis was as long as 133 and 176 days, respectively. These delays are likely due in part to a lack of awareness among radiologists and the non-specific, non-worrisome nature of most of these laboratory findings.

Half of the diagnostic work-ups were performed because of imaging or laboratory incidentalomas detected by blood tests or imaging carried out for other unrelated reasons. In this context, performing early diagnostic work-up in case of laboratory and imaging incidentalomas consistent with AE could be a critical measure for early AE diagnosis.

Serologic screening tests are known for their sensitivity in detecting AE, but our study found that 28.5% of results were false negative or inconclusive. This observation could be due to the initial use of outdated techniques (i.e. immunodiffusion using a triple concentrated serum with antigen from a soluble extract of alveolar cyst) or patient conditions (immunosuppression). Interestingly, WB showed a better sensitivity with positive results in three negative/inconclusive screening tests, highlighting the importance of this test in the laboratory diagnosis of AE.

In this study, the sensitivity of echo-endoscopic biopsy for the diagnosis of AE was low. Percutaneous biopsy was associated with better sensitivity.

Misdiagnosis was found to be common: In 72.5% (*n* = 16) of patients, AE was not part of the differential diagnosis suggested by the radiologists at the time of initial imaging.

One-third of patients who underwent surgery had an incorrect diagnosis of neoplasia at the time of surgery.

Diagnosis is often made at advanced disease stages: The median largest lesion size at diagnosis was 65 mm, with 54.5% of patients classified as stage IIIb or IV according to the WHO-IWGE classification. At the time of diagnosis, a significant proportion of patients had biliary (50%) or venous (36.5%) compression. Compared to the CHU Liège cohort, our patients were more likely to be diagnosed at a less severe stage [14]. In immunocompromised patients, larval EM infection causes a more rapid disease progression [41]. In our cohort, only two of seven immunocompromised patients had a disease stage higher than II. This may be explained by a higher number of incidentalomas (*n* = 6) in these patients, leading to earlier diagnosis. In one of the seven immunocompromised patients, disease progression was initially observed on imaging at the start of ABZ treatment.

In addition to the factors mentioned above, other elements are likely responsible for delayed diagnosis: unavailability of national guidelines for AE diagnosis; misclassification of AE due to cross-reactivity of first-line serology between *Echinococcus* species [42, 43]; failure of pathology examination to differentiate between AE and cystic echinococcosis [43]; diagnostic work-up requiring less available and more expensive imaging techniques such as MRI and PET.

### Treatment

While the WHO-IWGE guidelines recommend complete resection of the parasitic lesion with a safety margin, 32% of our patients were ineligible for radical surgery because of comorbidities or unresectable lesions due to metastases or vascular/biliary involvement. Only 54.5% of patients underwent curative intent surgery, of which only one-third underwent complete resection with a margin greater than 1 mm, as recommended.

Surgery is often complex and associated with high complication rates: In our cohort, 66.5% of patients undergoing curative intent surgery experienced postoperative complications. In this group, 62.5% had severe complications and one patient died.

In our study, as expected, a high proportion of patients experienced adverse effects attributed to ABZ, although only one was considered severe (liver enzymes increased more than tenfold). Underdosing and overdosing of ABZ were common, as shown by monitoring of ABZ sulfoxide plasma levels in our patients.

Regarding our observations, 85.5% of the patients showed either stable or reduced lesion size on imaging under ABZ treatment. However, in three patients (14%), disease progression was still observed at the start of ABZ treatment. One of them was immunocompromised and the ABZ sulfoxide plasma levels were not monitored; therefore, the possibility of drug underdosing could not be excluded. There was no apparent explanation for the other two patients, as they were non-immunosuppressed, treatment-compliant patients with initially elevated ABZ sulfoxide plasma levels that later normalized within the target range. Follow-up showed stability of lesion size after initial disease progression in two of these three patients. The third patient died during follow-up.

In addition to the factors mentioned above, other elements are likely to be responsible for the significant challenges in AE treatment: lack of national guidelines for AE treatment and follow-up; AE complications (biliary and vascular obstruction, pleural effusion, etc.) sometimes requiring expensive and complex interventional procedures or per-endoscopic interventions [30]; high frequency of ABZ shortages in Belgium due to stock issues, causing some patients to interrupt their treatment for weeks or even months; ABZ is a parasitostatic drug that requires long duration treatment, thus increasing the costs and risk of adverse effects; necessity of long-term imaging, laboratory, and clinical follow-up during and after ABZ treatment.

### Case detection

As early appropriate treatment significantly improves the prognosis of AE, diagnosing the disease at early stages could be the cornerstone for improving patient care and outcomes.

We highlighted interventions that could potentially improve case detection and diagnostic accuracy for AE: publication of national guidelines for the diagnosis and management of AE (finalized Belgian guidelines are expected to be published in early 2025); performing liver ultrasound screening in high-risk patients with occupational exposure [24]; adopting a low threshold for performing liver ultrasound in endemic regions for EM, especially in the presence of symptoms consistent with chronic inflammation or liver invasion; maintaining a low threshold for performing diagnostic work-up in incidentaloma cases, such as elevated liver enzymes or abnormal liver imaging; raising awareness among clinicians, including general practitioners (as already done in 2023 with a meeting for general practitioners in the Liège district), oncologists, gastroenterologists, radiologists, etc.; improving local expertise in AE diagnosis among radiologists, pathologists, surgeons, and infectious disease specialists in hospital networks in endemic areas; performing WB in patients with negative screening serology but high clinical and imaging suspicion of AE.

### Study strengths and weaknesses

Compared to previous Belgian cohort studies, our study tends to be more comprehensive in terms of clinical, laboratory, and patient management data [14, 16]. To our knowledge, EchiNam was the first Belgian study to identify and analyze the challenges and pitfalls of AE diagnosis and treatment in the country.

The main limitation of this study was its retrospective design and the limited number of patients.

For technical and organizational reasons, it was not possible to retrieve AE cases from the electronic medical records and laboratory databases of some hospitals between 2002 and 2011. As a result, some patients may have been missed.

Due to the study duration, continuous evolution of guidelines, and emergence of new diagnostic tools, some patients did not benefit from recent techniques, including WB, PCR, and monitoring of albendazole sulfoxide plasma levels, in the initial stages of their medical management.

Because of the evolution of PCR and serological methods and testing in different laboratories, the sensitivity and specificity of these tests may have changed over time.

## Conclusion

Although AE is a rare disease in Belgium, its prevalence and incidence are probably underestimated due to the long incubation period of the disease, misdiagnosis, and underreporting.

An efficient and resilient surveillance system is needed to obtain accurate and up-to-date epidemiological data on reservoirs (foxes and dogs) and human cases in Belgium.

Diagnosis is often made at advanced disease stages. Treatments then become more complex or even suboptimal, resulting in prolonged therapy, higher risk of adverse effects, significantly impaired quality of life, poor prognosis, and higher mortality rates.

Early diagnosis is crucial as it substantially improves the prognosis of AE if appropriate treatment is initiated promptly. Emphasis should be placed on interventions that have the potential to improve AE case detection and diagnostic accuracy, such as raising awareness among clinicians, screening programs, maintaining a low clinical suspicion threshold, and the use of sensitive diagnostic tests.

AE treatment is another important area of research, as there are currently no pharmaceutical alternatives to ABZ, which is often poorly tolerated.

## Electronic supplementary material

Below is the link to the electronic supplementary material.

Supplementary Material 1

## Data Availability

No datasets were generated or analysed during the current study.

## References

[1] Wen H, Vuitton L, Tuxun T, Li J, Vuitton DA, Zhang W et al (2019) Echinococcosis: advances in the 21st Century. Clin Microbiol Rev 32. 10.1128/CMR.00075-18 10.1128/CMR.00075-18 PMC6431127 30760475

[2] Blaser JBRDM, Mandell D, Bennett’s (2019) Principles and practice of infectious diseases, 9th edn. Elsevier - OHCE

[3] Wauters O, Honoré C, Detry O, Delwaide J, Demonty J, Léonard P et al (2005) [Alveolar Echinococcosis] Rev Med Liege 60:867–874 16402532

[4] Torgerson PR, Keller K, Magnotta M, Ragland N (2010) The global burden of alveolar echinococcosis. PLoS Negl Trop Dis 22(4):e722. 10.1371/journal.pntd.0000722 10.1371/journal.pntd.0000722 PMC2889826 20582310

[5] Deplazes P, Rinaldi L, Alvarez Rojas CA, Torgerson PR, Harandi MF, Romig T et al (2017) Global distribution of alveolar and cystic echinococcosis. Adv Parasitol 95. 10.1016/bs.apar.2016.11.001 10.1016/bs.apar.2016.11.001 28131365

[6] Vuitton DA, Demonmerot F, Knapp J, Richou C, Grenouillet F, Chauchet A et al (2015) Clinical epidemiology of human AE in Europe. Vet Parasitol 213. 10.1016/j.vetpar.2015.07.036 10.1016/j.vetpar.2015.07.036 26346900

[7] Romig T, Dinkel A, Mackenstedt U (2006) The present situation of echinococcosis in Europe. Parasitol Int. 10.1016/j.parint.2005.11.028 10.1016/j.parint.2005.11.028 16352465

[8] Hegglin D, Bontadina F, Deplazes P (2015) Human-wildlife interactions and zoonotic transmission of Echinococcus multilocularis. Trends Parasitol 31. 10.1016/j.pt.2014.12.004 10.1016/j.pt.2014.12.004 25599832

[9] Romig T (2009) Echinococcus multilocularis in Europe - State of the art. Vet Res Commun. 10.1007/s11259-009-9244-1 19578966 10.1007/s11259-009-9244-1

[10] Kern P, Bardonnet K, Renner E, Auer H, Pawlowski Z, Ammann RW et al (2003) European echinococcosis registry: human alveolar echinococcosis, Europe, 1982–2000. Emerg Infect Dis 9. 10.3201/eid0903.020341 10.3201/eid0903.020341 PMC2958541 12643830

[11] Gottstein B, Stojkovic M, Vuitton DA, Millon L, Marcinkute A, Deplazes P (2015) Threat of alveolar echinococcosis to public health–a challenge for Europe. Trends Parasitol 31:407–412. 10.1016/j.pt.2015.06.001 26115902 10.1016/j.pt.2015.06.001

[12] Delbecque K, Detry O, Hayette MP, Jeukens T, Delvenne P, Hardy N et al (2002) A case of hepatic alveolar echinococcosis contracted in Belgium. Acta Gastroenterol Belg 65 12014318

[13] Egrek S, Sacheli R, Detry O, Hayette M-P (2022) Activity report 2022 of the National Reference Laboratory for Echinococcosis

[14] Cambier A, Leonard P, Losson B, Giot JB, Bletard N, Meunier P et al (2018) Alveolar echinococcosis in southern Belgium: retrospective experience of a tertiary center. Eur J Clin Microbiol Infect Dis 37. 10.1007/s10096-018-3233-7 10.1007/s10096-018-3233-7 29564733

[15] Hanosset R, Saegerman C, Adant S, Massart L, Losson B (2008) Echinococcus Multilocularis in Belgium: prevalence in red foxes (Vulpes vulpes) and in different species of potential intermediate hosts. Vet Parasitol 14;151:212–217. 10.1016/J.VETPAR.2007.09.024 10.1016/j.vetpar.2007.09.024 18164551

[16] Detry O, Honoré C, Delwaide J, Demonty J, De Roover A, Vivario M et al (2005) Endemic alveolar echinococcosis in Southern Belgium? Acta Gastroenterol Belg 68 15832579

[17] Jansen F, Claes M, Bakkers E, Aryal A, Madimba KC, Gabriël S et al (2020) Echinococcus multilocularis in red foxes in North Belgium: prevalence and trends in distribution. Vet Parasitol Reg Stud Rep 22. 10.1016/j.vprsr.2020.100470 10.1016/j.vprsr.2020.100470 33308751

[18] Saitoh T, Takahashi K (1998) The role of vole populations in prevalence of the parasite (Echinococcus multilocularis) in foxes. Res Popul Ecol (Kyoto) 40. 10.1007/BF02765225

[19] Guislain MH, Raoul F, Giraudoux P, Terrier ME, Froment G, Ferté H et al (2008) Ecological and biological factors involved in the transmission of Echinococcus multilocularis in the French Ardennes. J Helminthol 82. 10.1017/S0022149X08912384 10.1017/S0022149X08912384 18394209

[20] Conraths FJ, Probst C, Possenti A, Boufana B, Saulle R, La Torre G et al (2017) Potential risk factors associated with human alveolar echinococcosis: systematic review and meta-analysis. PLoS Negl Trop Dis 11. 10.1371/journal.pntd.0005801 10.1371/journal.pntd.0005801 PMC5531747 28715408

[21] Cambier A, Giot JB, Leonard P, Bletard N, Meunier P, Hustinx R et al (2018) Multidisciplinary management of alveolar echinococcosis: Echino-Liege Working Group. Rev Med Liege 73 29595013

[22] McManus DP, Gray DJ, Zhang W, Yang Y (2012) Diagnosis, treatment, and management of echinococcosis. BMJ (Online) 344. 10.1136/bmj.e3866 10.1136/bmj.e3866 22689886

[23] Ammann RW, Eckert J, Cestodes (1996) Echinococcus. Gastroenterol Clin North Am 25. 10.1016/S0889-8553(05)70268-5 10.1016/s0889-8553(05)70268-5 8863045

[24] Piarroux M, Piarroux R, Giorgi R, Knapp J, Bardonnet K, Sudre B et al (2011) Clinical features and evolution of alveolar echinococcosis in France from 1982 to 2007: results of a survey in 387 patients. J Hepatol 55. 10.1016/j.jhep.2011.02.018 10.1016/j.jhep.2011.02.018 21354448

[25] Knapp J, Lallemand S, Monnien F, Felix S, Valmary-Degano S, Courquet S et al (2022) Molecular diagnosis of alveolar echinococcosis in patients based on frozen and formalin-fixed paraffin-embedded tissue samples. Parasite 29. 10.1051/parasite/2022004 10.1051/parasite/2022004 PMC8812296 35113014

[26] Knapp J, Lallemand S, Monnien F, Felix S, Courquet S, Umhang G et al (2023) Real-time multiplex PCR for human echinococcosis and differential diagnosis. Parasite 30. 10.1051/parasite/2023003 10.1051/parasite/2023003 PMC9886084 36700708

[27] Trachsel D, Deplazes P, Mathis A (2007) Identification of taeniid eggs in the faeces from carnivores based on multiplex PCR using targets in mitochondrial DNA. Parasitology 134. 10.1017/S0031182007002235 10.1017/S0031182007002235 17288631

[28] Brunetti E, Kern P, Vuitton DA (2010) Expert consensus for the diagnosis and treatment of cystic and alveolar echinococcosis in humans. Acta Trop 114. 10.1016/j.actatropica.2009.11.001 10.1016/j.actatropica.2009.11.001 19931502

[29] Caoduro C, Porot C, Vuitton DA, Bresson-Hadni S, Grenouillet F, Richou C et al (2013) The role of delayed 18F-FDG PET imaging in the follow-up of patients with alveolar echinococcosis. J Nucl Med 54. 10.2967/jnumed.112.109942 10.2967/jnumed.112.109942 23303963

[30] Vuitton DA, Azizi A, Richou C, Vuitton L, Blagosklonov O, Delabrousse E et al (2016) Current interventional strategy for the treatment of hepatic alveolar echinococcosis. Expert Rev Anti Infect Ther 14. 10.1080/14787210.2016.1240030 10.1080/14787210.2016.1240030 27686694

[31] Yang YR, Vuitton DA, Jones MK, Craig PS, McManus DP (2005) Brain metastasis of alveolar echinococcosis in a hyperendemic focus of Echinococcus multilocularis infection. Trans R Soc Trop Med Hyg 99. 10.1016/j.trstmh.2005.04.020 10.1016/j.trstmh.2005.04.020 16165174

[32] Liu W, Delabrousse É, Blagosklonov O, Wang J, Zeng H, Jiang Y et al (2014) Innovation in hepatic alveolar echinococcosis imaging: best use of old tools, and necessary evaluation of new ones. Parasite 21. 10.1051/parasite/2014072 10.1051/parasite/2014072 PMC4273719 25531446

[33] Bresson-Hadni S, Koch S, Miguet JP, Gillet M, Mantion GA, Heyd B et al (2003) Indications and results of liver transplantation for Echinococcus alveolar infection: an overview. Langenbecks Arch Surg 388. 10.1007/s00423-003-0394-2 10.1007/s00423-003-0394-2 12905036

[34] Zavoikin VD, Zelya OP, Tumolskaya NI (2021) Clinical tolerance and efficacy of anti-parasitic treatment with albendazole in patients with alveolar echinococcosis: long-term follow-up observation in 117 patients. Parasitol Res 120. 10.1007/s00436-021-07297-3 10.1007/s00436-021-07297-3 34432154

[35] Crouzet J, Grenouillet F, Delabrousse E, Blagosklonov O, Thevenot T, Di Martino V et al (2010) Personalized management of patients with inoperable alveolar echinococcosis undergoing treatment with albendazole: usefulness of positron-emission-tomography combined with serological and computed tomography follow-up. Clin Microbiol Infect 16. 10.1111/j.1469-0691.2009.02924.x 10.1111/j.1469-0691.2009.02924.x 19912267

[36] Arrêté royal du 18 septembre 2017 modifiant les annexes I et II de l’arrêté royal du 12 octobre 2004 fixant les conditions dans lesquelles l’assurance obligatoire soins de santé et indemnités intervient dans le coût des préparations magistrales et des produits assimilés

[37] Bresson-Hadni S, Piarroux R, Bartholomot B, Miguet JP, Mantion G, Vuitton DA (2005) Alveolar echinococcosis. EMC - Hepato-Gastroenterologie 2. 10.1016/j.emchg.2005.01.001 10.1002/hep.510270543 9581710

[38] Kern P, Wen H, Sato N, Vuitton DA, Gruener B, Shao Y et al (2006) WHO classification of alveolar echinococcosis: principles and application. Parasitol Int. 10.1016/j.parint.2005.11.041 16343985 10.1016/j.parint.2005.11.041

[39] Yu RY, Ellis M, Sun T, Li Z, Liu X, Vuitton DA et al (2006) Unique family clustering of human echinococcosis cases in a Chinese community. Am J Trop Med Hyg 74. 10.4269/ajtmh.2006.74.487 16525111

[40] Boulanger M, Léonard P, Egrek S, Detry O, Hayette MP (2023) Evaluation of the knowledge on alveolar echinococcosis among general practitioners in the province of Liege: impact of a formative intervention. Rev Med Liege 78 36924153

[41] Chauchet A, Grenouillet F, Knapp J, Richou C, Delabrousse E, Dentan C et al (2014) Increased incidence and characteristics of alveolar echinococcosis in patients with immunosuppression-associated conditions. Clin Infect Dis 59. 10.1093/cid/ciu520 10.1093/cid/ciu520 25034426

[42] Reiter-Owona I, Grüner B, Frosch M, Hoerauf A, Kern P, Tappe D (2009) Serological confirmatory testing of alveolar and cystic echinococcosis in clinical practice: results of a comparative study with commercialized and in-house assays. Clin Lab 55 19350848

[43] Stojkovic M, Mickan C, Weber TF, Junghanss T (2015) Pitfalls in diagnosis and treatment of alveolar echinococcosis: a sentinel case series. BMJ Open Gastroenterol 2. 10.1136/bmjgast-2015-000036 10.1136/bmjgast-2015-000036 PMC4599161 26462284

[44] Cisak E, Sroka J, Wójcik-Fatla A, ZajÄ.c V, Dutkiewicz J (2015) Evaluation of reactivity to Echinococcus spp. among rural inhabitants in Poland. Acta Parasitol 60. 10.1515/ap-2015-0074 10.1515/ap-2015-0074 26204192

[45] Knapp J, Sako Y, Grenouillet F, Bresson-Hadni S, Richou C, Gbaguidi-Haore H et al (2014) Comparison of the serological tests ICT and ELISA for the diagnosis of alveolar echinococcosis in France. Parasite 21. 10.1051/parasite/2014037 10.1051/parasite/2014037 PMC4111071 25058754

[46] Liance M, Janin V, Bresson-Hadni S, Vuitton DA, Houin R, Piarroux R (2000) Immunodiagnosis of Echinococcus infections: confirmatory testing and species differentiation by a new commercial western blot. J Clin Microbiol 38. 10.1128/jcm.38.10.3718-3721.2000 10.1128/jcm.38.10.3718-3721.2000 PMC87463 11015390

