# Supplementary material for: EchiNam: multicenter retrospective study on the experience, challenges, and pitfalls in the diagnosis and treatment of alveolar echinococcosis in Belgium
Source: Eur J Clin Microbiol Infect Dis. 2024 Nov 25;44(2):263–75. doi: 10.1007/s10096-024-04996-4 (PMC11754326; doi:10.1007/s10096-024-04996-4)
Supplement: Supplementary file 1 — Supplementary Material 1 [file 10096_2024_4996_MOESM1_ESM.docx]

**SUPPLEMENTARY DATA**


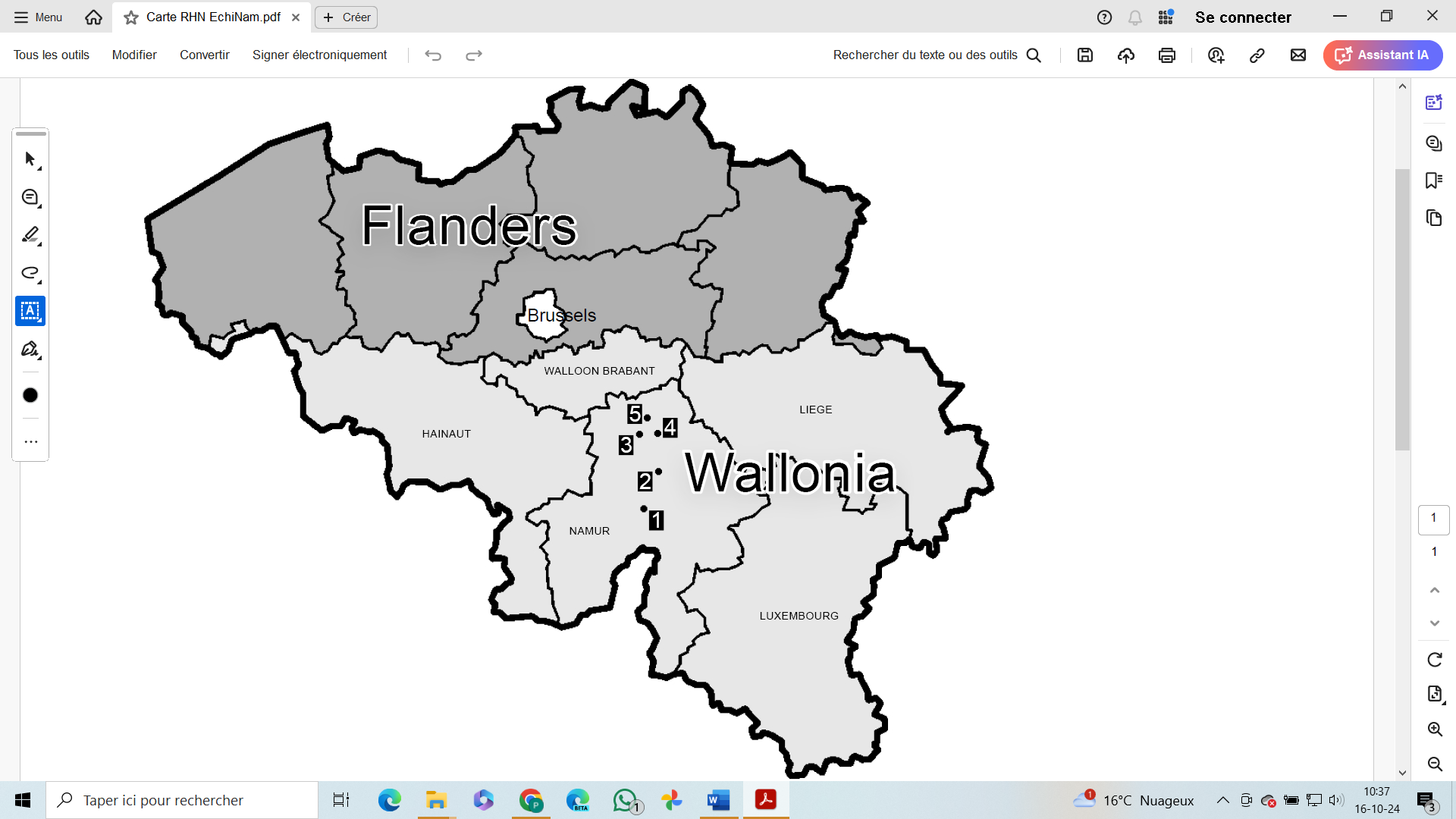


*Figure 1 Map of Belgium showing the Namur Hospital Network sites.*

*1: CHU UCL Namur site Dinant*

*2: CHU UCL Namur site Godinne*

*3: CHU UCL Namur site Namur*

*4: Centre Hospitalier Régional de Namur site Namur*

*5: Clinique Saint-Luc Bouge*

Practices of Belgian National Reference Laboratory for echinococcosis**:**

Currently, the Belgian National Reference Laboratory (BNRL) uses a two-step protocol for serological testing: screening by ELISA followed by confirmatory Western blot (WB).

The Em2plus ELISA test (Bordier) is used for screening with sensitivity and specificity greater than 90% [44,45].

WB is used for confirmation of a positive screening test, for differentiation between AE and cystic echinococcosis, and in case of high clinical suspicion, even if the screening test result is below the cut-off [46].

The BNRL uses a commercially available WB assay (which uses whole larval antigen extract from *E.* *multilocularis*) with a higher sensitivity (97%) than the screening ELISA tests [46].

The WB band pattern considered indicative of *Echinococcus* sp. infection includes 7 and/or 26-28 kDa bands. 16 and/or 18 kDa bands are considered specific for AE.

The BNRL currently uses two types of *Echinococcus* PCR: the first is a real-time multiplex PCR targeting the mitochondrial DNA gene (nad3) of EM together with the cox 2 gene specific for *Echinococcu*s *granulosus* (EG); the second is a nested PCR adapted from Trachsel *et al.* that is slightly more sensitive, targets mitochondrial genes (nad1, rrnS), and allows differentiation between EM and EG [27].

Real-time PCR was introduced into the BNRL’s diagnostic algorithm in 2022: Since then, real-time PCR is performed first, followed by nested PCR if negative.

The BNRL currently also performs sequencing of EM.
